# The Association of a Vegan Diet during Pregnancy with Maternal and Child Outcomes: A Systematic Review

**DOI:** 10.3390/nu16193329

**Published:** 2024-09-30

**Authors:** Deidre Meulenbroeks, Eline Otten, Sophie Smeets, Luuk Groeneveld, Daisy Jonkers, Simone Eussen, Hubertina Scheepers, Jessica Gubbels

**Affiliations:** 1 Department of Obstetrics & Gynaecology, GROW—Research Institute for Oncology and Reproduction, Maastricht University Medical Centre, P. Debyelaan 25, 6202 AZ Maastricht, The Netherlands; l.groeneveld@student.maastrichtuniversity.nl (L.G.); hcj.scheepers@mumc.nl (H.S.); 2 Department of Health Promotion, NUTRIM—School of Nutrition and Translational Research in Metabolism, Maastricht University, 6200 MD Maastricht, The Netherlands; eg.otten@student.maastrichtuniversity.nl (E.O.); slh.smeets@student.maastrichtuniversity.nl (S.S.); jessica.gubbels@maastrichtuniversity.nl (J.G.); 3 Department Gastroenterology-Hepatology, NUTRIM—School of Nutrition and Translational Research in Metabolism, Maastricht University, 6200 MD Maastricht, The Netherlands; d.jonkers@maastrichtuniversity.nl; 4 Department of Epidemiology, CARIM—Cardiovascular Research Insitute Maastricht, CAPHRI—School for Public Health and Primary Care, Maastricht University, 6200 MD Maastricht, The Netherlands; simone.eussen@maastrichtuniversity.nl

**Keywords:** plant-based diet, vegan, vegetarian, diet, pregnancy, systematic literature review, nutrition

## Abstract

** Background:** With the increasing prevalence of pregnant women adhering to a vegan diet, gaining insight into their nutritional intake and its association with maternal and fetal outcomes is essential to providing recommendations and developing guidelines for general practice. **Methods:** Following the Preferred Reporting Items for Systematic Reviews and Meta-Analyses (PRISMA) guidelines, a systematic review of the available scientific literature in Medline, Embase, and Cochrane was conducted in January 2024. **Results**: The titles and abstracts of 2211 unique articles were screened. Only six studies were eligible for inclusion and assessed for methodological quality using the (National Institutes of Health Study Quality (NIHSQ) Assessment Tool. The intake of protein and various micronutrients was significantly lower among vegan pregnant women compared to omnivorous women. Vitamin B12 supplements seemed sufficient in optimizing maternal and umbilical cord vitamin B12 levels amongst vegan mothers. Further, women on a vegan diet less often showed excessive pregnancy weight gain. However, children from women on a vegan diet had a significantly lower birth weight than those from women on an omnivorous diet. **Conclusion:** So far, only a few studies, with a large diversity of (assessment of) outcomes and insufficient power, have been published on this topic, limiting our ability to make firm conclusions about the effects of a vegan diet during pregnancy on maternal and fetal outcomes.

## 1. Introduction

The number of people on a vegan diet (also called a strict plant-based diet), which excludes the intake of all animal products such as meat and fish, dairy, eggs, and honey, is increasing [1]. Underlying reasons include ethical considerations, environmental concerns, and potential health benefits [2]. A survey conducted in various European countries in 2023 showed that between 1 and 8% of the population adhered to a vegan diet (2% in Scandinavian countries, 4% in the Netherlands and the United Kingdom, and 8% in Switzerland) [3]. Additionally, more than 75% of the vegan population is female and aged between 18 and 45 years, suggesting a higher prevalence of adhering to a vegan diet among fertile women [4,5].

Maternal prenatal nutrition is essential for maternal health and the unborn child’s health, emphasizing the importance of nutrition in the first one thousand days of life [6]. Maternal nutritional deficiencies during pregnancy may lead to serious complications [7,8]. For instance, maternal iron deficiency can lead to premature birth or low birth weight [9,10]. Further, iron deficiency is associated with maternal neuronal changes and problems in myelinization, neuronal transmission, and impaired frontal cortex and basal ganglia development [11]. Maternal iodine deficiency is also associated with aberrations in the neuronal development of children [12]. It can cause irreversible damage to the central nervous system, instilling permanent mental retardation [12]. Calcium deficiency increases the risk of maternal hypertensive disorders of pregnancy, osteopenia, prematurity, and low birth weight [13,14]. Newborns with a low level of selenium are at higher risk for developing retinopathy, bronchopulmonary dysplasia, and other lung disorders [15]. On the other hand, excess selenium can trigger cardiovascular problems, dyslipidaemias, and insulin resistance [15]. Lastly, macronutrients also have an important role in the health of the mother and child. Low maternal protein intake influences birthweight, and fatty acids impact the development of the brain and retina in the fetus and the risk of preeclampsia [14]. Animal foods, such as meat, dairy, and fish, contain many macronutrients and micronutrients. However, people on a vegan diet are also able to have a sufficient nutrient intake without consuming these animal foods, by consuming an abundance of plant foods such as grains, fruits, legumes, greens, nuts, and other fortified foods [16].

Current guidelines regarding a vegan diet during pregnancy are contradictory. The American Dietetic Association states that a well-planned vegan diet is appropriate for all life cycle stages, including pregnancy [16]. On the contrary, the German Nutrition Society advises against a vegan diet during pregnancy, lactation, and childhood due to the inadequate supply of essential nutrients, such as vitamin B12 [17]. The Dutch Nutritional Centre only advises women on a vegan diet to consult a dietician during pregnancy [18]. These guidelines have generally been based on the results of studies focusing on all types of vegetarian diets (including a vegan diet) in pregnancy [16,17]. However, while the vegetarian diet generally excludes the intake of meat and fish, it includes other animal products such as dairy and eggs, and is, therefore, not comparable to a vegan diet. This includes substantial differences between vegan and vegetarian diets in, for example, vitamin B12 and vitamin D intake, as these vitamins are available in dairy products and otherwise only in fortified vegan products or supplements [19,20]. Likewise, the few previous literature reviews that examined the effects of a vegan diet during pregnancy have included studies focusing on both vegan and vegetarian diets [7,21]. These reviews state that well-balanced vegetarian and vegan diets should be considered safe for the mothers’ health and offspring during pregnancy and lactation [7,21]. To develop consistent, evidence-based guidelines for women on a vegan diet during pregnancy, it is important to review the current evidence on the outcomes of women on exclusively a vegan diet in pregnancy. The aim of this review is to investigate the nutritional intake among pregnant women on a vegan diet and their maternal and fetal outcomes.

## 2. Materials and Methods

The systematic review was carried out following the Preferred Reporting Items for Systematic Reviews and Meta-Analysis (PRISMA) guidelines checklist [22] (Supplementary Table S1) and is registered in Prospero [CRD42022242456].

### 2.1. Data Sources and Search Strategies

Three electronic databases (PubMed, Embase, and Cochrane) were searched from 1 January 2000 to 24 January 2024. The electronic search strategies can be found in Appendix A. The search strategy consisted of different Mesh terms and keywords, combining terms related to vegan and vegetarian diets with terms for pregnancy. Even though this review does not focus on vegetarian diets, a vegan diet is sometimes referred to as a (type of) vegetarian diet in the literature and, therefore, the term vegetarian was included in the primary search. In addition, references of included studies and other systematic reviews were checked for additional relevant studies. The search results were transferred to Rayyan (Rayyan, 2023).

### 2.2. Eligibility Criteria

We included original studies describing the effects of a vegan diet in a population of pregnant women on maternal and/or fetal outcomes. Studies that focused on non-pregnant participants and studies with only participants with a non-vegan diet, e.g., pescatarians, flexitarians, lacto-vegetarians, ovo-vegetarians, and lacto-ovo-vegetarians (which all exclude some but not all animal-based foods) were excluded. Reviews and non-original studies, comments, editorials, letters to the editor, conference abstracts, case reports, articles without a full text available, and articles published before the year 2000 were excluded. No restriction was placed on language or outcome measures because of the expected limited available data.

### 2.3. Study Selection

Duplicates were removed. Titles and abstracts were screened for eligibility by two independent reviewers (DM with either EO, SS, or JG). Full texts of articles that potentially contained results about pregnant women on a vegan diet were retrieved. If these were not available online, authors or journals were contacted. Two authors decided independently on final inclusion and exclusion based on full texts (DM with either EO, SS, or JG). In case of discrepancies between authors, another independent check of the original publication was performed by a third person. After discussion, the authors made a decision on inclusion or exclusion.

### 2.4. Data Collection, Extraction, and Synthesis

Two individual authors (DM with either EO or SS) conducted the data extraction; data were combined, and in case of differences between authors, another check of the original publication was performed. The corresponding author of the included articles was contacted for further information when data were unclear or not provided in the article. This review describes all outcomes assessed in the included studies comparing vegan versus omnivorous diets and all subgroup analyses within these groups. Reported subgroups with other diets in the included articles are out of the scope of the current review. A meta-analysis was not planned because of the expected limited number of studies on this topic and the diversity of the outcomes assessed.

### 2.5. Risk of Bias

The included studies were assessed for methodological quality using the National Institutes of Health Study Quality Assessment Tool for Observational Cohort and Cross-sectional studies [23]. This tool consists of 14 items, assessing the clarity of the research question, the participation rate of the eligible persons, the sample size justification, and whether confounding variables were measured and adjusted for statistically, among others. Possible answers were ‘Yes’, ‘No’, and ‘Other’ (Cannot Determine, Not Reported, and Not Applicable). Two individual authors (DM and JG) independently assessed each study. Results were combined, and if differences appeared between authors, a decision on the rating was made after discussion. Total scores were categorized into good quality, fair quality, or poor quality, depending on ratings of the criteria.

## 3. Results

### 3.1. Study Selection

**Figure 1:**
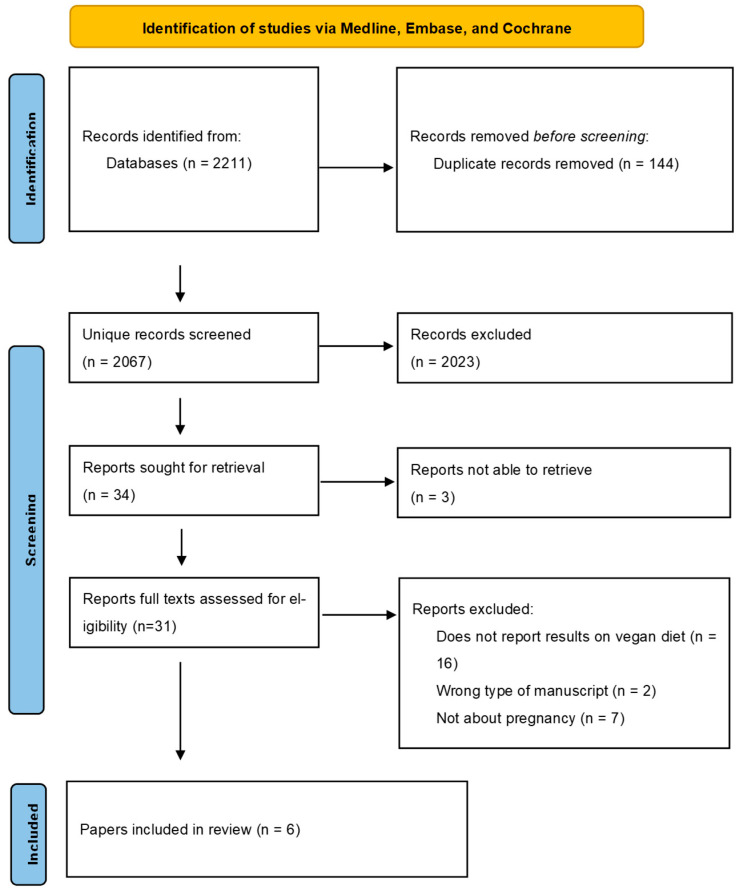
Prisma Flow diagram.

A total of 2211 studies were identified during the search. After removing duplicates, 2067 unique articles were screened based on title and abstract. Finally, six articles were included in this systematic review. The search results can be found in the Prisma Flow diagram (Figure 1).

### 3.2. Study Characteristics

The study characteristics of the six included publications can be found in Table 1. All studies [24,25,26,27,28,29] included vegan and omnivore participants; they also all included a vegetarian group, but those results are not discussed in this review. Two publications [24,25] were prospective cohort studies based on the same group of participants from a prospective cohort study; the other four publications were based on four different cross-sectional studies [26,27,28,29]. The studies by Avnon et al. and Kesary et al. were performed in Israel [24,25,26], the study by Ferrara et al. was performed in Italy [27], the study by Hedegaard et al. was conducted in Denmark [28], and the study by Pawlak et al. was conducted in the USA [29]. All six included publications reported on fetal outcomes [24,25,26,27,28,29], and four, additionally, reported on maternal outcomes [24,25,26,28].

### 3.3. Risk of Bias within Studies

The risk of bias assessment of the included studies can be found in Table 2. All studies had a clear research question [24,25,26,27,28,29]. Only the publication by Avnon and Anbar presented a sample size justification [24]. Sample sizes ranged from 18 to 234 women on a vegan diet and 15 to 65,872 women on an omnivorous diet. Kesary et al. only included women with a stable dietary pattern, defined as a vegan diet for more than one year prior to conception [26]. Avnon and Anbar et al. and Avnon and Dubinsky et al. only enrolled women who maintained the same diet for at least 3 months prior to and throughout the current pregnancy [24,25]. Hedegaard et al. and Pawlak et al. did not mention the diet duration for inclusion in the study [28,29]. Ferrara et al. described how long women were on a specific diet, but did not use this as an enrollment criterion [27]. In all studies, dietary assessment was briefly described. Ferrara et al. and Hedegaard et al. [27,28] used a validated food frequency questionnaire to assess the participants’ diet [30,31]. In contrast, the other studies used a self-developed survey with questions regarding mothers’ dietary adherence during pregnancy [24,25,26,29]. After assessing the relevance of every subtopic, the overall methodological quality of the publication by Avnon and Anbar et al. was categorized as ‘good’ [24], that of Hedegaard et al. and Kesary et al. as ‘fair’ [26,28], and that of the other studies as ‘poor’ [25,27,29].

### 3.4. Results of Individual Studies

The main results are listed in Table 3 and Table 4. The most important results concerning the mother and fetus are discussed.

#### 3.4.1. Maternal Nutrient Intake

One study reported on macro nutrient intake [28] and showed a significantly lower protein intake in vegan women than in omnivorous women (*p* < 0.05). No significant differences were found concerning the total energy, fat, carbohydrate, and fiber intake [28]. The intake of several micronutrients (retinol, vitamin B12, vitamin D, calcium, and iodine) was significantly lower in vegan women compared to omnivorous women (iodine *p* < 0.01, others *p* < 0.05) [28]. On the other hand, beta-carotene and folate intake were significantly higher in the vegan group (*p* < 0.01) [28]. The iron intake was not significantly different comparing vegan women with omnivorous women [28]. When women’s dietary supplement intake was added to the dietary contribution, the median intake of vitamin A, folic acid, vitamin B12, vitamin D, calcium, iron, and iodine was well above the Danish recommended nutrient intake for most omnivores [28]. This was also the case for most vegans, except for the median vitamin D intake of 6.2 μg/day (recommended level 10 μg/day) [28].

#### 3.4.2. Maternal Plasma Concentrations of Nutrients

One publication reported on maternal plasma concentrations of nutrients [24]. Avnon and Anbar et al. found no difference in plasma ferritin, hemoglobin, vitamin B12, and folic acid levels among pregnant women on a vegan diet compared to omnivores [24]. However, subanalysis showed a significantly higher circulating vitamin B12 levels in women on a vegan diet who took multivitamins in combination with iron supplements compared to women on a vegan diet with no multivitamins or iron supplementation (388.29 pg/mL ± 209.54 vs. 219.63 pg/mL ± 95.26, *p* = 0.03) [24]. This difference between women who did or did not take supplements was not seen in the omnivore group [24].

#### 3.4.3. Maternal Weight Gain

Two studies reported on maternal weight gain [25,26]. They both showed a significantly lower pregnancy weight gain in pregnant women on a vegan diet compared to women with an omnivorous diet, with a 1.60 kg (*p* = 0.002) [26] and 2.66 kg (*p* < 0.001) [25] lower weight gain, respectively. Additionally, Kesary et al. found significantly fewer women with excessive weight gain during pregnancy (defined as more than 18 kg for normal-weight women pre-pregnancy and more than 12 kg for women who were overweight pre-pregnancy) amongst women on a vegan diet compared to omnivores, also after adjustment for age and pre-pregnancy BMI (*p* = 0.004) [26].

#### 3.4.4. Maternal Pregnancy-Related Outcomes

Three publications reported on maternal pregnancy-related outcomes [25,26,28]. Hedegaard et al. showed a significantly higher prevalence of pre-eclampsia in pregnant women on a vegan diet compared to an omnivorous diet (*p* < 0.05) [26], while Avnon and Dubinsky et al. showed no significant differences in hypertensive complications [25]. There were no significant differences (all *p*-values >0.05) between women on a vegan and those on an omnivorous diet in the prevalence of gestational diabetes mellitus [25,26,28], iron deficiency before week 30 [28], preterm birth [28], induction of labor [28], cesarean section [28], or postpartum hemorrhage [25].

#### 3.4.5. Umbilical Cord Nutrient Levels

One publication reported on umbilical cord nutrient levels [24]. Avnon and Anbar et al. found that no significant differences were observed for umbilical cord levels of hemoglobin, ferritin, vitamin B12, and folic acid between vegan and omnivorous mothers, nor was the prevalence of anemia or nutrient deficiency significantly different between infants of mothers on a vegan diet versus those on an omnivorous diet [24]. Subgroup analysis showed a significantly higher vitamin B12 measurement in the umbilical cord blood of women on a vegan diet who took multivitamins and iron supplements (1002.63 pg/mL ± 608.56 vs. 442.57 pg/mL ± 151.30, *p* < 0.001) compared to vegan women without the use of multivitamins and iron supplements [24]. This difference was not observed in the omnivorous group (*p* > 0.05) [24].

#### 3.4.6. Birth Weight

Five publications reported on birth weight [25,26,27,28,29]. Several studies showed a significantly lower birth weight in grams [25,27], lower birth weight centile [26,27], and/or higher number of small-for-gestational-age (SGA) children [25] in children born from mothers on a vegan diet compared to children born from mothers on an omnivorous diet. However, other studies reported no significant differences in birth weight centile [28,29], number of SGA children [26,28], or number of children with low birth weight (<2500 g) [26,28,29] in children born from vegan mothers compared to children born from omnivorous mothers.

## 4. Discussion

To the best of our knowledge, this systematic review is the first study to specifically examine the associations of a vegan diet during pregnancy with maternal and fetal outcomes. This review showed that a limited number of studies have been published on this topic, with serious limitations that must be considered when interpreting the results.

It was noticed that many publications report about pregnant vegetarian women while also including vegan women, even though there are significant differences between the nutritional content of both diets. Data about vegan pregnant women specifically were not always described, nor were they available after contacting the authors of such publications combining data on vegan and vegetarian participants. Further, the group of vegan women studied was often relatively small. As various articles on potentially relevant data sets had to be excluded due to the focus on all types of vegetarian women combined, this review included only six publications [24,25,26,27,28,29]. It is important to take note of the possible risk of bias in the included studies. Sample size justification is fundamental in observational cohorts and cross-sectional studies to ensure a study has enough participants to detect an association, if one truly exists [23]. Most of the included studies were not designed to be sufficiently powered to answer the prespecified questions about a vegan diet [25,26,27,28,29]. This could be explained by the focus on vegetarian diets compared to the omnivorous diet in most included studies, with a vegan diet as a small subgroup. With Kesary et al. [26] being the exception, most studies included a limited number of vegan women (<*n* = 60) and did not all adjust for all other factors that could influence both maternal and fetal outcomes, such as family history, obstetrical history, parity, maternal BMI, and the use of potentially relevant medication. Also, the studies did not report the participation rate of eligible women, probably because participants were recruited online or via group emails. Responding bias could have influenced the self-reported outcomes. Given the poor quality of some of the studies, the results found in the included studies should be viewed as hypothesis-generating and not as definite conclusions.

The significantly lower vitamin B12 intake among vegan mothers compared to omnivore mothers and the sufficient intake of the majority of both groups considering combined nutritional intake from foods and supplements aligns with previous studies about the positive effect of vitamin B12 suppletion in women with vitamin B12 deficiencies in pregnancy [32,33]. Even though vegan foods, such as plant-based milk, are increasingly fortified with vitamin B12, the limited current data available indicate that supplement usage should be promoted in women on a vegan diet. In addition, the pregnant women’s median intake of vitamin D was frequently below the recommended level, independent of the diet. Therefore, the Dutch Health Council advises all Dutch pregnant women to supplement vitamin D with 10 mcg per day, as it lowers the risk for an SGA neonate or asthma-like symptoms independent of diet [34]. Additionally, Hedegaard et al. described that when dietary supplements were added to the dietary contribution, the median intake of iron, folic acid, calcium, iron, and iodine was well above the recommended nutrient intake for most participants, independent of their diet. However, it should be noted that their study was based on nutrient intake in 1996–2002, a period in time where supplement intake and food intake were substantially different compared to the current time, given the recent increase in the availability of fortified foods and different supplementation behavior and recommendations.

Notably, studies found a lower fetal birth weight among children born to mothers on a vegan diet compared to mothers on an omnivorous diet [25,26,27]. Results regarding the incidence of SGA were mixed, with one study showing a larger incidence [25] and others not showing a significant difference [26,28]. A low birth weight is an unfavorable outcome, as it is a major contributor to the development of cardiovascular diseases later in life [35]. According to the Barker hypothesis, suboptimal nutrition in intrauterine life includes a functional and structural change in the fetus, subsequently leading to various illnesses, such as hypertension, diabetes, and obesity [36]. Therefore, promoting adequate nutritional intake in women before and during pregnancy may be a promising strategy for preventing chronic diseases worldwide [37]. Hedegaard et al. suggest that the lower birthweight among children of women on a vegan diet could be due to the lower protein intake in their group of vegan pregnant women [28]. Compared to the current recommended intake of protein for pregnant women in Denmark (10–20% of energy, corresponding to 0.8–1.5 g protein/kgbodyweight/day), nearly half of the vegan mothers had an insufficient protein intake, with a mean intake of 10.4% per day [28]. Exploring the possible influence of protein intake, including the difference between animal-based versus plant-based proteins and their bioavailability and biofunctionality, on birthweight in future studies with a larger sample size is recommended.

The prevalence of excessive gestational weight gain (GWG) in pregnancy was lower amongst vegan women compared to omnivore women [26]. Excessive GWG (above the guidelines) is associated with a higher risk of pregnancy-induced hypertension, gestational diabetes, large-for-gestational-age babies (LGA), macrosomia, and cesarean section [38,39]. On the other hand, GWG below guidelines (no studies in included publications) is associated with a higher risk for SGA babies and preterm birth compared to GWG within guidelines [40]. The focus should, therefore, be on appropriate GWG, as recommended by the Institute of Medicine [40].

One study found a higher incidence of pre-eclampsia among vegan women in pregnancy [28], while another study found no differences in the incidence of hypertensive disorders among vegan women compared to omnivorous women in pregnancy [25]. Pre-eclampsia affects between 3 and 5% of pregnant women in the general population [41]. There are different risk factors for the development of pre-eclampsia, such as prior pre-eclampsia, chronic hypertension, pregestational diabetes, multifetal pregnancy, antiphospholipid syndrome, pre-pregnancy obesity, systemic lupus erythematosus, nulliparity, and maternal age over 40 years. Nutritional status and dietary intake of key foods and nutrients can also influence the pre-eclampsia risk [42,43]. On the other hand, aspirin is a preventive drug treatment for pre-eclampsia, and a daily calcium intake of >1 g reduces the rates of pre-eclampsia [41]. Most of the factors influencing the risk of pre-eclampsia were not described in either of the included publications. It is, therefore, unknown if the incidence of pre-eclampsia was affected by the diet of the pregnant women. Other maternal outcomes, such as the prevalence of gestational diabetes mellitus and iron deficiency, were not found to be significantly different amongst vegan women compared to omnivorous women in the current review.

### 4.1. Strengths and Limitations

Our study had several strengths. To our knowledge, it is the first systematic review focusing on specifically a vegan diet in pregnancy compared to an omnivorous diet. The search strategy was extensive, focusing on terms related to vegan and vegetarian diets, as a vegan diet is sometimes referred to as a vegetarian diet. Additionally, corresponding authors were contacted for further information when data was unclear or missing, e.g., whether vegans were included in a study about pregnant vegetarian women.

Our study also had some limitations. Three databases were used to find publications on a vegan diet in pregnancy, and studies published on other platforms can be overlooked. However, references to included studies and other systematic reviews were checked for additional relevant studies to make sure relevant studies would not be missed. Because of the low number of included studies published on a vegan diet in pregnancy, studies were not excluded because of the risk of bias assessment. No meta-analyses, tests for heterogeneity or sensitivity analyses were performed. Given the generally poor quality of some of the included studies, the results found in this review should be viewed as hypothesis-generating and not as definite conclusions.

### 4.2. Recommendations Future Research

Studies about a vegan diet in pregnancy with more participants in a sufficiently powered case-control study would provide more knowledge about nutritional intake and maternal and fetal outcomes. A large cohort study with enough power to detect an association between nutrition and maternal and fetal outcomes does not seem feasible, given the relatively low prevalence of women adhering to a vegan diet. As vegan participants in studies about vegetarian diets in pregnancy are currently scarce, it would be interesting to report results separately for different subtypes of vegetarian diets, including the vegan diet. Nutritional intake and supplements, as well as medical and obstetrical history, medication use, and lifestyle habits, should also be considered in analyzing the association of a vegan diet with maternal and child outcomes. The intake of different dietary products and the duration of a specific diet can significantly affect the mother’s nutritional intake [44]. It is, therefore, important to keep track of participants’ diets and intake of macro- and micronutrients to compare future study results and acquire more available data on this topic. Using validated food questionnaires and the healthful and unhealthful plant-based diet index could help address these issues [45,46].

## 5. Conclusions

Current existing guidelines for pregnant women on a vegan diet have been mainly based on studies among vegetarians, sometimes including vegans, and expert opinions. This systematic review on a vegan diet, specifically, showed that children from mothers on a vegan diet seem to have a lower birth weight and, hence, a higher risk for SGA compared to children from mothers on an omnivorous diet. This may be related to a low protein intake and warrants further study. Furthermore, although dietary intake of various micro-nutrients was insufficient, using vitamin B12 supplements seems to be sufficient in optimizing maternal and umbilical cord vitamin B12 levels among vegan mothers. However, too few studies with inadequate sample sizes based on the current nutritional intake have been published to draw conclusions about the effects of a vegan diet during pregnancy on maternal and fetal outcomes.

## Figures and Tables

**Table 1:** Study characteristics.

Study			Methods		Results		
Author	Year	Country	Study Design	Data Collection	Participants ^1,2^	Primary Outcome	Secondary Outcome
Avnon and Anbar [24]	2020	Israël	Prospective cohort	Maternal and umbilical cord blood tests, questionnaire	60 vegans 37 pescatarians 64 vegetarians 112 omnivores	Maternal cord levels: vit B12, folic acid, ferritin, hemoglobin, Umbilical cord levels: Vit B12, folic acid, ferritin, hemoglobin	Deficiencies in maternal and umbilical cord blood levels according to diet Blood levels vit B12 according to oral supplementation
Avnon and Dubinsky [25]	2020	Israël	Prospective cohort	Questionnaire	60 vegans 37 pescatarians 64 lacto/ovo-vegetarians 112 omnivores	Preterm birth (<37 weeks) Incidence of small for gestational age.	Birthweight, gestational diabetes, pre-eclampsia, gestational hypertension, PPH, gestational weight gain, 5-min APGAR score below 7
Ferrara [27]	2019	Italy	Cross-sectional study	Online questionnaire	21 vegans 19 vegetarians 15 omnivores	Birth weight Birth length Birth cranial circumference	-
Hedegaard [28]	2024	Denmark	Prospective cohort study	Computer-assisted telephone interviews, food frequency questionnaire	18 vegans 666 pescatarians 183 lacto/ovo-vegetarians6 5872 omnivores	Dietary composition and quantity of consumed energy and macro- and micronutrients (energy, protein, fat, carbohydrates, fiber, retinol, beta-carotene, folic acid, vitamin B12, vitamin D, calcium, iron, iodine)	Pregnancy outcomes (birthweight, birth length, gestational age, low birth weight (<2500 g), small for gestational age, male infants, spontaneous delivery, induced labor, cesarean section, iron deficiency before week 30, gestational diabetes, preeclampsia, gestational age)
Kesary [26]	2020	Israël	Cross-sectional study	Online questionnaire posted on social media	234 vegans 133 vegetarians 1052 omnivores	Maternal: Excessive weight gain, gestational diabetes mellitus Fetal: small for gestational age (birth weight <10th centile), large for gestational age (birth weight >90 centile), preterm delivery (<37 weeks)	Maternal: weight gain during gestation Fetal: birth weight centile, low birth weight <2500 g
Pawlak [29]	2014	USA	Cross-sectional study	Online questionnaire sent to Seventh-Day Adventist Church schools and churches, announcement on web-based blog	47 vegans 199 vegetarians 350 omnivores	Birth weight difference prevalence of low birth weight (<2500 g)	Prevalence of low birth weight (2500 g)

^1^ all participants were women; among the participants in all studies were also vegetarians and/or pescatarians; their outcomes are not reported in this review. ^2^ Definitions: omnivores: people with a dietary pattern in which food of both plant and animal origin are included; vegans: people with a dietary pattern in which foods of animal origin are totally excluded; vegetarians: people with a dietary pattern that excludes meat, meat-derived foods, and, to different extents, other animal products; pescatarians: people with a dietary pattern that excludes meat and meat-derived foods, but includes fish; lacto/ovo-vegetarians: people with a dietary pattern that excludes meat and meat-derived foods, but includes eggs and dairy products.

**Table 2:** The risk of bias in the included studies as assessed using the National Institutes of Health Study Quality Assessment Tool for Observational Cohort and Cross-sectional studies [15].

Author (Year)	Clear Research Question	Population Specified and Defined	Participation Rate of Eligible Persons >50%	All Subjects Similar Populations	Sample Size Justification	Exposure (s) of Interest Measured Prior to the Outcome (s)	Timeframe Sufficient to See Association	Did the Study Examine Different Levels of Exposure?	Exposure Measures Clearly Defined, Valid, Reliable, Implemented Consistently	Exposure Assessed More than Once	Outcome Measures Clearly Defined, Valid, Reliable, Implemented Consistently	Outcome Assessors Blinded to Exposure Status of Participants	Loss to Follow-Up <20%	Confounding Variables Measured and Adjusted Statistically	Quality Rating (Good, Fair, Poor)
Avnon and Anbar [24]	Yes	Yes	NR	Yes	Yes	Yes	Yes	NA	Yes	No	Yes	NR	NR	No	Good
Avnon and Dubinsky [25]	Yes	Yes	NR	Yes	NR	Yes	Yes	NA	Yes	No	Yes	NR	NR	Yes	Poor
Ferrara [27]	Yes	Yes	NR	Yes	NR	Yes	Yes	NA	Yes	No	Yes	NR	NR	No	Poor
Hedegaard [28]	Yes	Yes	No	Yes	NR	Yes	Yes	NA	Yes	No	Yes	NR	NR	Yes	Fair
Kesary [26]	Yes	Yes	NR	Yes	NR	Yes	Yes	NA	Yes	No	Yes	NR	NR	Yes	Fair
Pawlak [29]	Yes	Yes	NR	No	NR	Yes	Yes	NA	Yes	No	Yes	NR	NR	No	Poor

NA = not applicable; NR = not reported.

**Table 3:** Maternal outcomes.

Author	Outcome Measure(s)	Summary Main Findings ^a^	Significance ^b^
Avnon and Anbar [24]	Maternal blood Hemoglobin (g/dL) Ferritin (ng/mL) Vit B12 (pg/mL) Folic acid (ng/mL) Deficiencies in blood levels Maternal anemia (n (%)) Maternal vit B12 deficiency (n (%))	Maternal blood 12.24 ± 1.04 vs. 12.39 ± 1.08 27.71 ± 17.37 vs. 34.26 ± 44.97 361.37 ± 204.76 vs. 325.84 ± 151.53 15.55 ± 3.32 vs. 36.52 ± 2.47 Deficiencies 6 (10.00) vs. 9 (8.04) 10 (17.54) vs. 10 (9.26)	Maternal blood NS NS NS NS Deficiencies NS NS
Avnon and Dubinsky [25]	Preterm birth (<37 weeks) (n (%)) GDM (n (%)) Hypertensive complications (n (%)) Postpartum hemorrhage (n (%)) Gestational weight gain (mean kg SD) *	5 (3) vs. 4 (3.57) 5 (8.33) vs. 10 (8.93) 2 (3.33) vs. 2 (1.79) 3 (5) vs. 10 (8.93) 11.65 ± 4.22 vs. 14.31 ± 4.57	NS NS NS NS *p* < 0.001
Hedegaard [28]	Macronutrients (mean (SD)) Energy (MJ/day) Protein (%E) Fat (%E) Carbohydrates (%E) Fiber (g/day) Micronutrients (median (10th–90th percentile)) Retinol (μg/day) Beta-carotene (μg/day) Folic Acid (μg/day) Vitamin B12 (μg/day) Vitamin D (μg/day) Calcium (g/day) Iron (mg/day) Iodine (μg/day) Micronutrients relative to recommended intake (median (% above lower boundary)) Vitamin A IU, recommended 800 Folic acid (μg/day), recommended 500 Vitamin B12 (μg/day), recommended 2 Vitamin D (μg/day), recommended 10 Calcium (g/day), recommended 0.9 Iron (mg/day), recommended 15 Iodine (μg/day), recommended 200 Pregnancy outcomes Spontaneous delivery (n (%)) Induced labor (n (%)) Cesarean section (n (%)) Iron deficiency before week 30 (n (%)) Gestational diabetes (n (%)) Preeclampsia (n (%))	Macronutrients 9.7 (2.5) vs. 10.4 (2.8) 10.4 (2.6) vs. 15.4 (2.4) 32.7 (5.7) vs. 32.3 (6.1) 56.7 (6.1) vs. 51.8 (5.9) 30 (11) vs. 27 (9) Micronutrients 419 (224–1146) vs. 645 (323–1219) 2.9 (1.1–1.6) vs. 2.1 (0.9–5.2) 379 (224–588) vs. 350 (237–498) 1.5 (0.4–6.9) vs. 6.4 (3.5–10.2) 1.1 (0.3–5.5) vs. 3.3 (1.7–6.3) 0.9 (0.5–2.1) vs. 1.4 (0.8–2.1) 11.5 (7.8–17.0) vs. 11.4 (7.8 vs. 15.7) 222 (117–312) vs. 268 (162–413) Micronutrients relative to recommended intake (food + supplements) 1326 (72) vs. 1414 (90) 599 (61) vs. 623 (67) 9.4 (89) vs. 10.4 (>99) 6.2 (39) vs. 10.2 (51) 1.2 (72) vs. 1.5 (91) 35 (89) vs. 63 (92) 332 (72) vs. 381 (94) Pregnancy outcomes 12 (66.7) vs. 44,421 (67.9) 3 (16.7) vs. 20,870 (32.1) 1 (5.6) vs. 9917 (15.3) 1 (5.6) vs. 4993 (7.7) 0 (0.0) vs. 512 (0.6) 2 (11.1) vs. 1680 (2.6)	Macronutrients NS *p* < 0.05 NS NS NS Micronutrients *p* < 0.01 *p* < 0.01 *p* < 0.01 *p* < 0.01 *p* < 0.01 *p* < 0.01 NS *p* < 0.05 Recommended intake NA NA NA NA NA NA NA Pregnancy outcomes NS NS NS NS NS *p* < 0.05
Kesary [26]	Excessive weight gain (n (%)) Adjusted odds ratio (95% CI) ** GDM (n (%)) Adjusted odds ratio (95% CI) *** Weight gain gestation (mean kg SD)	56 (24.7) vs. 348 (35.6) 0.61 (0.44–0.86) 11 (4.7) vs. 86 (8.2) 0.59 (0.31–1.14) 12.2 ± 5.7 vs. 13.8 ± 5.8	*p* < 0.001 *p* = 0.004 NS NS *p* = 0.002

GDM: gestational diabetes mellitus; NS: not significant; NA: not available. ^a^ vegans compared to omnivores unless stated otherwise. ^b^ vegans compared to other groups: lacto-ovo-vegetarians, fish-eaters, omnivores unless stated otherwise. * adjusted to age and pre-pregnancy BMI. ** adjusted for maternal age, birth week, smoking status, GDM, and risk [adjusted odds ratio (aOR)] of EWG. *** adjusted for age, smoking, and pre-pregnancy BMI. Nordic Nutrition Recommendation 2023 recommendations for pregnant women, only the iron value for lactating women was used.

**Table 4:** Fetal outcomes.

Author	Outcome Measure(s)	Summary Main Findings ^a^	Significance ^b^
Avnon and Anbar [24]	Umbilical Hemoglobin (g/dL) Ferritin (ng/mL) Vit B12 (pg/mL) Folic acid (ng/mL) Deficiencies in blood levels Umbilical anemia (n (%)) Umbilical ferritin deficiency (n (%)) Umbilical vit B12 deficiency (n (%)) Umbilical folic acid deficiency (n (%))	Umbilical 15.56 ± 2.21 vs. 15.76 ± 2.23 199.02 ± 120.87 vs. 233.26 ± 216.80 902.05 ± 586.60 vs. 778.57 ± 402.04 20.51 ± 4.66 vs. 20.61 ± 5.01 Deficiencies 4 (8.33) vs. 5 (5.10) 3 (7.32) vs. 12 (14.12) 2 (5.00) vs. 1 (1.23) 0 (0) vs. 1 (1.67)	Umbilical NS NS NS NS Deficiencies NS NS NS NS
Avnon and Dubinsky [25]	SGA (n (%)) * Birthweight (gram SD) * 5-min Apgar score below 7 (n (%))	7 (11.67) vs. 2 (1.79) 3015.2 ± 420.4 vs. 3328 ± 495.8 0 (0) vs. 0 (0)	*p* = 0.018 *p* = 0.002 *p* < 0.001
Ferrara [27]	Birth weight Birth length Birth head circumference	Lower in grams Lower in percentiles No difference No difference	*p* = 0.03 ^a^ *p* = 0.02 ^a^ NS ^a^ NS ^a^
Hedegaard [28]	Birth weight (mean g (SD)) Birth length (mean cm (SD)) Gestational age (mean days (SD)) Low birth weight <2500 g (n (%)) SGA (n (%)) Male infants (n (%))	3441 ± 558 vs. 3601 ± 544 52.5 ± 2.6 vs. 52.3 ± 2.6 285.6 ± 9.9 vs. 280.6 ± 11.9 2 (11) vs. 1623 (2.5) 5 (27.8) vs. 6502 (9.9) 7 (38.9) vs. 33,792 (51.3)	NS NS *p* < 0.01 *p* < 0.05 NS NS
Kesary [26]	SGA (n (%)) Adjusted odds ratio (95% CI) ** LGA, (n (%)) Adjusted odds ratio (95% CI) ** preterm delivery <37 weeks (n (%)) Birthweight centile (mean p (SD)) Low birth weight <2500 g (n (%))	24 (10.3) vs. 67 (6.4) 1.59 (0.95–2.65) 13 (5.6) vs. 100 (9.5) 0.60 (0.33–1.11) 6 (2.6) vs. 46 (4.4) 42.6 ± 25.9 vs. 52.5 ± 27.0 10 (4.3) vs. 57 (5.4)	NS NS NS NS NS *p* < 0.001 NS
Pawlak [29]	Birth weight (Mean kg (SD)) Prevalence of low birth weight (%)	3.54 (0.51) vs. 3.32 (0.63) 0% vs. 7.1%	NS NS

SGA: small for gestational age (birth weight <10th centile); LGA: large for gestational age (birth weight >90th centile); NS: not significant. ^a^ vegan compared to omnivores unless stated otherwise. ^b^ vegan compared to other groups: lacto-ovo-vegetarian, fish-eaters, and omnivores unless stated otherwise. * adjusted to age and pre-pregnancy BMI. ** adjusted for age, birth week, smoking, and pre-pregnancy BMI.

## Supplementary Materials

The following supporting information can be downloaded at: https://www.mdpi.com/article/10.3390/nu16193329/s1, Table S1: Prima 2020 Checklist.

## Appendix A

**Electronic search strategies** **Search Pubmed:** ((“Vegetarians”[MeSH] OR “Diet, Vegetarian”[MeSH] OR “Vegans”[MeSH] OR “Diet, Vegan”[MeSH] OR vegan*[tiab] OR vegetarian*[tiab] OR plant-base*[tiab] OR plantbase*[tiab])) AND (((“Pregnancy Outcome”[Mesh] OR maternal outcome*[tiab] OR “Diabetes, Gestational”[Mesh] OR “Hypertension, Pregnancy-Induced”[Mesh] OR “Pre- Eclampsia”[Mesh] OR “HELLP Syndrome”[Mesh] OR “Anemia”[Mesh] OR “Fetal Growth Retardation”[Mesh] OR “Fetal Macrosomia”[Mesh] OR “Infant, Low Birth Weight”[Mesh] OR “gestational diabetes*”[tiab] OR “high blood pressure*”[tiab] OR pre-eclampsia*[tiab] OR preeclampsia*[tiab] OR toxemia*[tiab] OR macrosomia*[tiab] OR anemia*[tiab] OR anaemia*[tiab]) OR (fetal*[tiab] OR fetus*[tiab] OR foetus*[tiab] OR embryo*[tiab]) AND (grow*[tiab] OR size*[tiab] OR develop*[tiab] OR outcome*[tiab]) OR (ctal*[tiab] OR newborn*[tiab] OR birth*[tiab]) AND (size*[tiab] OR weight*[tiab] OR birthweight[tiab] OR develop*[tiab] OR outcome*[tiab])) OR ((intra-uterine[tiab] OR intrauterine[tiab]) AND (grow*[tiab] OR develop*[tiab]))) **Search Embase:** (exp vegetarian/or exp vegetarian diet/or exp vegan diet/OR exp vegan/OR vegan*.ti,ab,kw. OR vegetarian*.ti,ab,kw. OR plant-base*.ti,ab,kw. OR plantbase*.ti,ab,kw.) AND (exp pregnancy outcome/OR exp pregnancy diabetes mellitus/OR exp maternal hypertension/OR exp hellp syndrome/OR exp preeclampsia/OR exp”eclampsia and preeclampsia”/OR exp anemia/OR exp intrauterine growth retardation/OR exp macrosomia/OR exp low birth weight/OR maternal outcome*.ti,ab,kw. OR “pregnancy diabetes melitus”*.ti,ab,kw. OR hypertensi*.ti,ab,kw. OR “high blood pressure”*.ti,ab,kw. OR pre-eclampsia*.ti,ab,kw. OR preeclampsia*.ti,ab,kw. OR toxemia*.ti,ab,kw. OR macrosomia*.ti,ab,kw. OR anemia*.ti,ab,kw. OR anaemia*.ti,ab,kw. OR ((fetal*.ti,ab,kw. OR fetus*.ti,ab,kw. OR foetus*.ti,ab,kw. OR embryo*.ti,ab,kw.) AND (grow*.ti,ab,kw. OR size*.ti,ab,kw. OR develop*.ti,ab,kw. OR outcome*.ti,ab,kw.)) OR ((neonatal*.ti,ab,kw. OR newborn*.ti,ab,kw. OR birth*.ti,ab,kw.) AND (size*.ti,ab,kw. OR weight*.ti,ab,kw. OR birthweight*.ti,ab,kw. OR develop*.ti,ab,kw. OR outcome*.ti,ab,kw.)) OR ((intra-uterine.ti,ab,kw. OR intrauterine.ti,ab,kw.) AND (grow*.ti,ab,kw. OR develop*.ti,ab,kw.))) **Search Cochrane:** (MeSH descriptor: [Vegans] explode all trees OR MeSH descriptor: [Vegetarians] explode all trees OR MeSH descriptor: [Diet, Vegan] explode all trees OR MeSH descriptor: [Diet, Vegetarian] explode all trees OR vegan* OR vegetarian* OR plant- base* OR plantbase*) AND MeSH descriptor: [Pregnancy Outcome] explode all trees OR MeSH descriptor: [Diabetes, Gestational] explode all trees OR MeSH descriptor: [Hypertension, Pregnancy-Induced] explode all trees OR MeSH descriptor: [Pre-Eclampsia] explode all trees OR MeSH descriptor: [HELLP Syndrome] explode all trees OR MeSH descriptor: [Anemia] explode all trees OR MeSH descriptor: [Fetal Growth Retardation] explode all trees OR MeSH descriptor: [Fetal Macrosomia] explode all trees OR MeSH descriptor: [Infant, Low Birth Weight] explode all trees OR gestational diabetes* OR high blood pressure* OR pre- eclampsia* OR preeclampsia* OR toxemia* or macrosomia* OR anemia* OR anaemia* OR ((fetal* OR fetus* OR foetus* OR embryo*) AND (grow* OR size* OR develop* OR outcome*)) OR ((neonatal* OR newborn* OR birth*) AND (size* OR weight* OR birthweight* OR outcome*)) OR ((intra-uterine* OR intrauterine*) AND (grow* OR develop*)
